# Highly Porous Polyimide Gel for Use as a Battery Separator with Room-Temperature Ionic Liquid Electrolytes

**DOI:** 10.3390/gels12020108

**Published:** 2026-01-27

**Authors:** Rocco P. Viggiano, James Wu, Daniel A. Scheiman, Brianne DeMattia, Patricia Loyselle, Baochau N. Nguyen

**Affiliations:** 1 NASA Glenn Research Center, 21000 Brookpark Road, Cleveland, OH 44135, USA; james.j.wu@nasa.gov (J.W.);; 2 Universities Space Research Association, 425 3rd Street SW, Suite 950, Washington, DC 20024, USA

**Keywords:** polyimide, polyimide aerogel, polyimide gel separator, room-temperature ionic liquids, ionic conductivity

## Abstract

Advanced aerospace vehicle concepts demand concurrent advances in energy storage technologies that improve both specific energy and safety. Commercial lithium-ion batteries commonly employ polyolefin microporous separators and carbonate-based liquid electrolytes, which can deliver room-temperature ionic conductivities on the order of 10^−3^–10^−2^ S/cm but rely on inherently flammable solvents. Room-temperature ionic liquids (RTILs) offer a nonvolatile, nonflammable alternative with a stable electrochemical window; however, many RTILs exhibit poor compatibility and wetting with polyolefin separators. Here, we evaluate highly porous, cross-linked polyimide (PI) gel separators based on 4,4′-oxydianiline (ODA) and biphenyl-3,3′,4,4′-tetracarboxylic dianhydride (BPDA), cross-linked with Desmodur N3300A, formulated with repeating unit lengths (n) of 30 and 60. These PI gel separators exhibit an open, fibrillar network with high porosity (typically >85%), high thermal stability (onset decomposition > 561 °C), and high char yield. Six imidazolium-based RTILs containing 10 wt% LiTFSI were screened, yielding nonflammable separator/electrolyte systems with room-temperature conductivities in the 10^−3^ S/cm range. Among the RTILs studied, 1-ethyl-3-methylimidazolium bis(trifluoromethylsulfonyl)imide (EMIM-TFSI) provided the best overall performance. Ionic conductivity and its retention after four months of storage at 75 °C were evaluated in the EMIM-TFSI/LiTFSI system, and the corresponding gel separator exhibited a tensile modulus of 26.66 MPa. Collectively, these results demonstrate that PI gel separators can enable carbonate-free, nonflammable RTIL electrolytes while maintaining the ionic conductivity suitable for lithium-based cells.

## 1. Introduction

A separator is a critical component used in batteries. It is a membrane that maintains a fixed distance between the anode and cathode while permitting ionic transport. It is fabricated to be as thin as possible to ensure minimal contribution to the overall cell mass, thereby maximizing the specific energy achievable by the cell. Ideally, battery separators for lithium-based batteries should possess the following properties: chemical stability, a thickness of 1 mil or less, at least 40% porosity, mechanical strength, wettability for chosen electrolytes, and dimensional stability [1,2].

To date, inexpensive and commonly used separators are composed of polyolefin such as Celgard^®^ (low melting point ~130 °C), used in lithium-ion batteries [3,4]. Celgard^®^ separators range in thickness from 20 to 25 µm. Polyolefin separators are typically used with electrolyte compositions including ethylene carbonate (EC), dimethyl carbonate (DMC), or propylene carbonate (PC) solvents suitable for both dissolving the lithium salt and wetting the polyolefin separator [5]. This separator/electrolyte combination exhibits ionic conductivities ranging from 1 × 10^−3^ S/cm to 1 × 10^−2^ S/cm at room temperature. However, polyolefin separators have melting points below the thermal runaway temperature of lithium-ion batteries, poor thermal stability, and inherent flammability [6]. Efforts to improve the performance of separators by reducing their flammability and shrinkage, while improving ionic conductivity, have been intensively investigated [7].

Several polymer substrates like polyacrylonitrile (PAN) and its composites [8,9,10] have been of interest due to their higher porosity (>68%) and higher mechanical strength than most Celgard^®^ separators (porosity > 45%) [1]. Other materials explored include poly(vinylidene fluoride) [11,12], copolymer poly(methyl methacrylate)-poly(vinylidene fluoride) [13], poly(m-phenylene isophthalamide) [14], poly(arylene ether nitrile) [15], and the polyvinyl butyral (PVB)–urethane network [16], for instance. While these materials exhibit improved properties, the carbonate-based electrolytes or wetting solvents still impose a flammability risk [6].

Room-temperature ionic liquids (RTILs) are another class of electrolytes considered for improving battery safety. RTILs are salt-like liquids composed of organic cations and inorganic or organic anions. Depending on the cation/anion chemistry, RTIL viscosities can approach ~860 cP at 20 °C [17,18]. They can exhibit high ionic conductivity (up to 10^−2^ S/cm) and, more notably, are known for their negligible volatility [19,20] and thermal stability above ~200 °C to 400 °C [21,22]. However, it has been found that several RTILs have poor wettability on polyolefin separators at the electrolyte/separator interface, lowering the effective ionic conductivity [23,24]. Carbonate co-solvents such as ethylene carbonate or propylene carbonate have therefore been used to improve RTIL wetting [25,26].

Contact angle measurements for separators with improved wetting properties for RTILs have been widely investigated [23,24,27,28]. Research on electrospun polyacrylonitrile microfiber (PAN) separators has shown better wetting with 1-methyl-1-propylpyrrolidinium bis(trifluoromethylsulfonyl)imide (PYR13-TFSI) or 1-methyl-1-propylpyrrolidinium bis(fluorosulfonyl)imide (PYR13-FSI) (in 1:1 ethylene carbonate and diethyl carbonate) compared to Celgard polypropylene (PP) separators, due to their higher porosity, 83% vs. 39%, respectively [24]. A study conducted by Caimi et al. [27] showed an improvement in the contact angle when SiO_2_ nanoparticles (10 wt%) were incorporated into poly(vinylidene fluoride-co-hexafluoropropylene) (PVDF-HFP) in PYR1308b, and when sodium dodecyl sulfate was used as a wetting agent. The type of anion used, such as bis(trifluoromethanesulfonyl)imide (TFSI^−^) or tetrafluoroborate (BF_4_^−^), also contributes to RTIL uptake [29]. Cheruvally et al. [29] demonstrated that electrospun poly(vinylidene fluoride-co-hexafluoropropylene), PVDF-HFP, membranes could absorb 750% of 1-butyl-3-methylimidazolium (BMI) with TFSI^−^ and 600% of BMI with BF_4_^−^. Both systems exhibited similar conductivities of 2.3 × 10^−3^ S/cm at 25 °C. The ability of the PVDF-HFP membrane to retain a large volume of BMI was attributed to its high surface area with an average fiber diameter of <1 µm, which helped to reduce electrolyte leakage.

Over the last decade, interest in separators with high porosity and high surface area has expanded to aromatic polyimide aerogels. Numerous studies have focused on the synthesis and development of polyimide aerogels, having improved mechanical strength up to 200 MPa [30,31] and attaining excellent thermal stability with onsets of decomposition ranging from 460 to 610 °C [30,31,32]. These attributes substantially exceed those of commonly used polyolefin separators (e.g., the industry-standard Celgard®), which exhibit markedly lower thermal stability and dimensional integrity at elevated temperaturesPolyimide aerogels are an interesting choice as a porous scaffold that serves as both a battery separator and an electrolyte reservoir due to their high porosity (85–95%) and smaller pore sizes [33]. This also improves the suppression of lithium dendrites [33]. In addition, the high degradation temperature of polyimide aerogels could, in principle, improve battery performance at elevated temperatures, above 300 °C, for long durations [33,34,35], thereby decreasing flammability and thermal runaway risks [34,36]. With a wide selection of monomers, the backbone structures of aromatic polyimide aerogels can be tailored to make flexible or rigid substrates [30,34] of varying thicknesses with tunable hydrophobicity and wettability to suit different types of electrolytes, ILs in particular [33]. Nevertheless, the electrochemical properties of these cross-linked polyimide aerogels have typically been evaluated using electrolytes based on ILs in mixtures of diethyl carbonate and ethylene carbonate [33,34,35,36].

In our previous study, the pore structures of a polyimide (PI) gel and its corresponding polyimide aerogel, composed of the diamine 4,4′-oxydianiline (ODA), the dianhydride biphenyl-3,3′,4,4′-tetracarboxylic dianhydride (BPDA), and the triisocyanate cross-linker Desmodur N3300A, were investigated using small-angle neutron scattering (SANS) [37]. The experiment was performed on a polyimide aerogel and on polyimide gels solvated with H_2_O, D_2_O, and three different RTILs (1-ethyl-3-methylimidazolium bis(trifluoromethylsulfonyl)imide, 1-methyl-1-propylpyrrolidinium bis(trifluoromethylsulfonyl)imide, and 1-butyl-1-methylpyrrolidinium bis(trifluoromethylsulfonyl)imide). The data confirmed the collapse of some of the polyimide aerogel struts and pores during the supercritical drying process, leading to a lower volume fraction of solvent in the solvated polyimide aerogels when compared to the solvent-exchanged gels.

The focus of our study is to develop a novel battery separator type based on highly porous polyimide gels (ODA/BPDA/N3300A) that are compatible with several room-temperature ILs, resulting in ionic conductivities of up to 10^−3^ S/cm. Two N3300A cross-linked polyimides with repeating units n of 30 and n of 60 were fabricated. The characterization of these ODA/BPDA/N3300A polyimide aerogels (dry state) was evaluated using solid-state ^13^C nuclear magnetic resonance (^13^C NMR), Fourier Transform Infrared spectroscopy (FTIR), and thermogravimetric analysis (TGA) in N_2_. The micro- and nano-porous structure of the PI aerogel was captured using scanning electron microscopy (SEM). Their BET surface areas, pore sizes, and pore volumes were also analyzed. Based on the SANS results [37], six selected imidazolium-based ILs (Table 1) with 10 wt% lithium bis(trifluoromethanesulfonyl)imide (Li salt) were screened for ionic conductivities, with results up to 10^−3^ S/cm. The thickness of the highly porous and tortuous polyimide gel framework imbibed with the most effective electrolyte, 1-ethyl-3-methylimidazolium bis(trifluoromethylsulfonyl)imide (EMIM-TFSI) with Li salt on the ionic conductivity (IC) was examined. Ionic conductivities were measured at room temperature and 75 °C to assess temperature dependence. The tensile properties of this separator/electrolyte were performed. The replacement of the TFSI (CF_3_SO_2_)_2_N^−^ anion with the tetrafluoroborate (BF_4_^−^) anion on their IC was also examined.

## 2. Results and Discussion

**Scheme 1:**
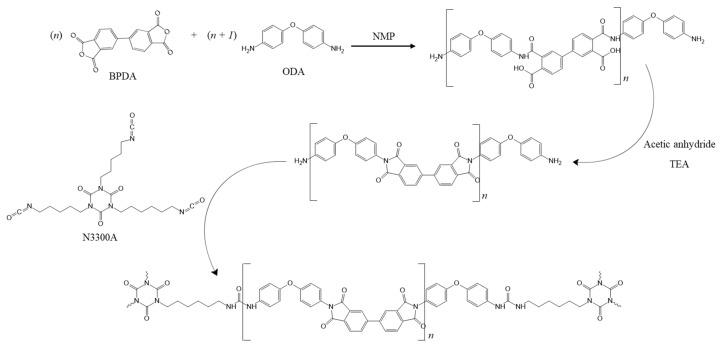
Mechanism for ODA-BPDA based polyimide gel, cross-linked with N3300A (Ref. [37]).

The physical, thermal, and morphological properties of ODA/BPDA/N3300A polyimide (PI) gel and its corresponding polyimide aerogel have been well studied [30,37]. The mechanism for the polyimide reaction is presented in Scheme 1 [37]. The chemical structure of the PI aerogel with *n* of 30, verified via solid-state ^13^C nuclear magnetic resonance (NMR), is shown in Figure S1 and the Fourier Transform Infrared Spectroscopy (FTIR) is shown in Figure S2.

**Figure 1:**
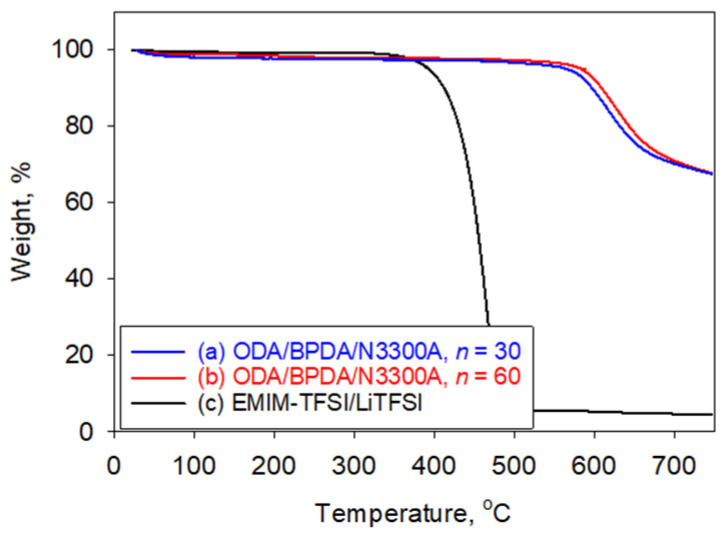
TGA of (a) ODA/BPDA/N3300A aerogel with *n* = 30, (b) ODA/BPDA/N3300A aerogel with *n* = 60, and (c) EMIM-TFSI/LiTFSI (10 wt% Li salt) in N_2_.

Plotted in Figure 1a,b are the thermogravimetric analysis (TGA) traces of the cross-linked PI aerogels formulated with *n* = 30 and *n* = 60, respectively, collected in N_2_. An initial weight loss of approximately 1–2% near 100 °C was attributed to desorption of moisture and/or residual volatiles. The formulation with lower *n* (higher N3300A content) exhibited a subtle mass loss feature near 250 °C, consistent with the decomposition of the aliphatic segments in the cross-linker [38,39]. The cross-linked PI aerogels with *n* = 30 and *n* = 60 showed onset decomposition temperatures (T_d_) of 561 °C and 567 °C, respectively, with high char yields (67.6%).

**Figure 2:**
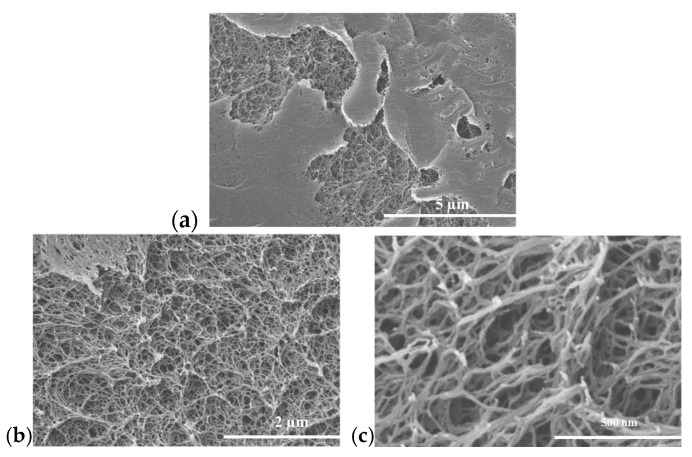
SEM images of (**a**) formation of thin skin on the top surface of the PI aerogel film, and pore structures of the PI aerogel underneath the skin layer at different magnifications: (**b**) 2 µm and (**c**) 500 nm.

During the film casting process, the exposed top surface of the gel pores partially collapsed and formed a thin skin on the outer surface. In addition, during the supercritical drying process, the PI aerogel underwent some shrinkage due to a reduction in the polymer struts [37], further densifying the film surfaces. Figure 2a shows the SEM image of the aerogel film (*n* = 60), highlighting the differences between the top-side skin and the pore structure underneath the film surface. Figure 2b,c (at higher magnification) reveal the retained pore structure beneath the skin layer. The BET surface areas and porosities measured for PI aerogels synthesized in this study were 419 m^2^/g and 91% for *n* = 30, and 344 m^2^/g and 87% for *n* = 60. Accordingly, depending on the film thickness and the processing, a PI aerogel separator can exhibit different pore volumes and thus different capacities to serve as an ionic liquid reservoir (Figure S3).

**Figure 3:**
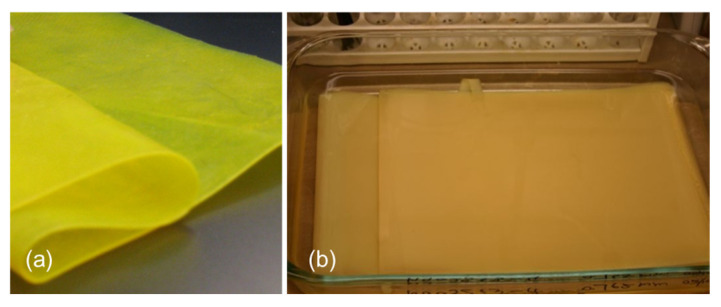
Pictures of (**a**) the PI aerogel film after supercritical drying and (**b**) the PI gel film in EMIM-TFSI/Li salt with an *n*-value of 60.

Displayed in Figure 3a is the PI aerogel film after supercritical drying and Figure 3b shows the PI gel film (with *n* of 60) in EMIM-TFSI/Li salt electrolyte solution. The cast PI films were very flexible and foldable.

The hierarchical pore structure formed in the ODA/BPDA/N3300A aerogel comprises an interconnected fibrillar network with micro- and nano-porous pathways that can facilitate ion transport when the gel is solvated with an ionic liquid electrolyte. In our prior work, the ODA/BPDA/N3300A aerogel was shown to have lower porosity than the corresponding solvent-exchanged gel [37]. In the present study, we examine how the nano-porous structure in the PI gel separator influences ionic conductivity in RTIL/LiTFSI electrolytes.

The imidazolium-based ILs were selected based on viscosity and ionic conductivity, as well as reported electrochemical and thermal stability [23,37]. The viscosities ranged from 27.9 cP to 56.0 cP, and ionic conductivities ranged from 2.6 mS/cm to 14.1 mS/cm (measured at 20–30 °C) (Table 1). Table 2 summarizes ten experimental runs using PI gel films formulated with *n* = 30 and *n* = 60 at different final film thicknesses after solvent exchange into ILs containing 10 wt% Li salt, with ionic conductivity measured at 25 °C. ILs with higher viscosity required longer times to fully imbibe the gel films (e.g., ~2 days for ILs B and C), whereas lower-viscosity ILs typically imbibed within ~1 day.

The synthetic route and process to produce the polyimide gel and polyimide aerogel are reported in the experimental section. The complete removal of acetone after the solvent exchange with ILs was verified by TGA. Graphed in Figure 1c is the TGA plot of 1-ethyl-3-methylimidazolium bis(trifluoromethylsulfonyl)imide (EMIM) (IL-E) with 10 wt% Li salt after acetone removal. No weight loss attributable to acetone was observed. Only ~0.5 wt% weight loss occurred between 110 °C and 325 °C before the first onset of the decomposition of EMIM at 370 °C, perhaps due to residual moisture from the Li salt when exposed to air during the TGA preparation.

**Figure 4:**
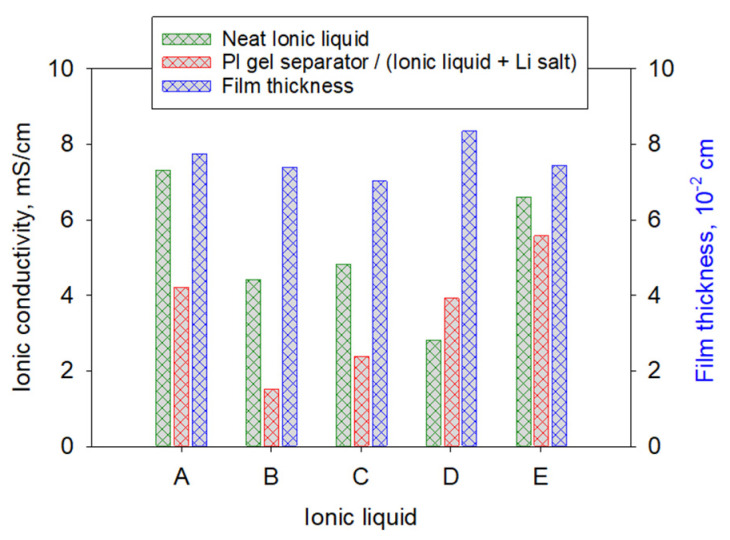
Comparison of neat ILs and PI gel separator with IL + Li salt and the gel film thickness measured at room temperature (25 °C). Specimens were from the same batch of gel film cast with the same initial thickness and *n* = 30 (runs 1–5).

The ionic conductivities (IC) of the polyimide gel films with *n* = 30, cast from the same batch, and imbibed with five different ionic liquids A–E (runs 1–5) were tested, as listed in Table 2. These ionic liquids had the same TFSI anion, (CF_3_SO_2_)_2_N^−^, for comparison. Therefore, the differences in ionic conductivities arise mainly from changes in cation interactions with the polyimide gel separator. The commonly used LiTFSI salt (Li salt) was chosen for this study. Figure 4 is a bar chart representing the IC of the neat ILs (data obtained from IoLiTec, Inc. technical data sheets), the measured ICs of the polyimide (PI) gel separator with IL/Li salt systems, and the corresponding final thicknesses of the polyimide gel film separators. It should be mentioned that once fully imbibed with IL/Li salt (or electrolyte), the polyimide gel separators swelled to different thicknesses, suggesting that they absorbed different amounts of IL/Li salt within their pores and skeletal framework, depending on the chemical structure of the ILs [33]. It should be noted that the polyimide gel separators were prepared in the solvated state. Prior to the exchange of IL/Li salt, the gel films were imbibed with acetone. Therefore, the initial thickness of a gel film solvated in acetone was not measured to determine the % electrolyte uptake.

In general, the ionic conductivity of an IL decreases when a Li salt is added, consistent with most ILs tested here and with prior reports [23,28]. An exception was observed for IL-D (run 4), which showed an increase in ionic conductivity upon addition of Li salt. IL-D is reported to have a relatively low viscosity (27.9 cP) and high ionic conductivity (14.1 mS/cm) in its neat form (Table 1), which may partially offset the salt-induced conductivity loss. Across the ILs examined, the magnitude of conductivity reduction varied (Table 2), suggesting that specific ion–polymer interactions, ion pairing, and/or microstructural effects within the PI gel influence effective transport in the IL/LiTFSI-solvated separators.

**Table 2:** Screening results of ionic conductivity of polyimide gels with different repeating unit, *n*, and thicknesses in various ILs.

Run #	RTIL Type	*n*	Ionic Conductivity of RTIL/Li Salt Solvated PI Gel (mS/cm)	Loss in Ionic Conductivity (%)	Film Thickness of RTIL Solvated PI Gel (cm)
1 ^a^	A	30	4.19	(−) 42.4	0.0774
2 ^a^	B	30	1.49	(−) 66.2	0.0738
3 ^a^	C	30	2.37	(−) 50.7	0.0702
4 ^a^	D	30	3.91	(+) 41.7	0.0834
5 ^a^	E	30	5.57	(−) 16.0	0.0742
6 ^b^	E	60	3.23	(−) 51.3	0.0158
7 ^b^*	E	60	2.84	(−) 57.2	0.0158
8 ^c^	E	60	2.54	(−) 61.7	0.0087
9 ^c^	E	60	1.69	(−) 74.5	0.0036
10 ^c^	F	60	3.76	(−) 73.3	0.0076

^a^ From same batch of film cast with *n* = 30. ^b^ From same batch of film cast with *n* = 60. ^b^* Ionic conductivities measured 4 months after preparation. ^c^ From same batch of film cast with *n* = 60.

Based on the ionic conductivity screening results (Table 2), polyimide gel separators imbibed with IL-E/Li salt provided the best combination of ionic conductivity and electrochemical stability. To quantify the sensitivity of conductivity to key variables, the following factors were evaluated using Design-Expert 12: repeating-unit length (*n*), final gel thickness after IL/Li salt exchange, and the conductivity of the room-temperature IL/Li salt solvated PI gel. The resulting regression model captured the observed trends (R^2^ = 0.9451; standard deviation = 0.3935) and indicated that film thickness and effective pore morphology (including the presence of a surface skin layer) are dominant contributors to measured ionic conductivity in these PI gel separators.

**Figure 5:**
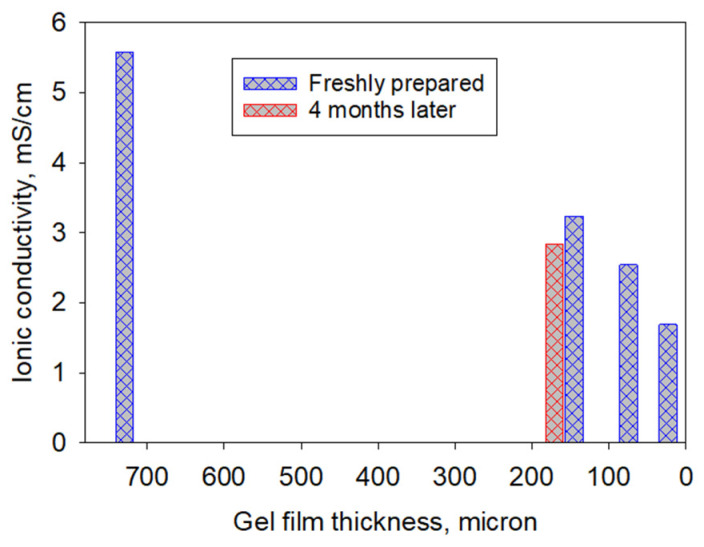
Ionic conductivity of IL-E/Li salt in polyimide gel separators of different thicknesses (blue bars) and corresponding IC on a PI gel separator after 4 months (red bar) (R^2^ = 0.9451, std. dev. = 0.3935). Data were analyzed using Design Expert software.

All data were analyzed using Stat-Ease software Design-Expert 12. The results suggested that the separator thickness had a significant effect on ionic conductivity (R^2^ = 0.9451, standard deviation = 0.3935), as shown in Figure 5, whereas the backbone chain length (*n*) did not. As the thickness of the gel film decreased from 745 µm to 36 µm, a drop in IC was observed, from (−)16% down to (−)74.45% (Table 2, runs 5–9). The skin that formed on top of the film (Figure 3a,b) may have contributed to this drop as a result of the partial blockage of ion transport between the two electrodes. Note that the ion-transporting channels between these separators can change as a function of surface area and porosity.

An additional experiment measured the ionic conductivity of a separator (158 µm) imbibed with IL-E/ Li salt after four months of storage. A modest decrease in ionic conductivity was observed, from 3.23 mS/cm (run 6) to 2.84 mS/cm (run 7). This change is consistent with minor electrolyte compositional changes during storage (e.g., moisture uptake) and is within the scatter expected for thickness and interfacial–contact uncertainties in EIS measurements.

The effect of temperature on the ionic conductivity of the PI gel film/separator (*n* of 60) imbibed with IL-E/Li salt, was screened at room temperature (RT) and at 75 °C. After the initial IC measured at RT, the separator, 0.0126 cm thick, was heated at 75 °C for 48 h. An increase in IC from 2.23 mS/cm to 3.42 mS/cm was observed. The higher IC obtained at an elevated temperature implied that the PI gel itself is stable in the presence of the electrolyte, while higher temperature aided the distribution of the electrolyte within the PI gel porous structure. Their IES plots are demonstrated in Figure S4.

The mechanical properties of the PI gel film with *n* of 60 and thickness of 0.0158 cm, imbibed with IL-E/Li salt (sample 6) was performed. The average tensile stress at maximum load was 0.61 ± 0.03 MPa and tensile modulus was 26.66 ± 0.78 MPa average. Results obtained were listed in Table S1. Variation in tensile values may have been due to the amount of the electrolyte solution retained in the samples during the test.

**Figure 6:**
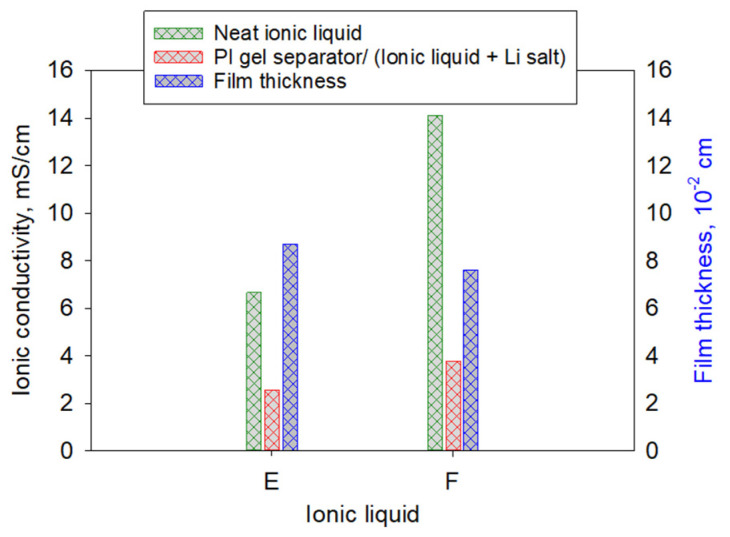
Comparison of IL-E (Run #8) and IL-F (Run #10) and their corresponding PI gel separators with electrolyte, and gel film thickness measured at room temperature (25 °C). Specimens were prepared from the same batch of gel film cast with the same initial thickness and *n* of 60.

Similarly to IL-E, which uses bis(trifluoromethylsulfonyl)imide (TFSI^−^) as the anion, IL-F incorporates tetrafluoroborate (BF_4_^−^) and is of interest due to its relatively high ionic conductivity and low viscosity [22,36]. As summarized in Table 1 and Figure 6, IL-F exhibits the highest neat-IL conductivity among the ILs examined. When incorporated into the PI gel separator, IL-F (run 10) yielded a higher measured conductivity than IL-E (run 8), 3.76 mS/cm versus 2.54 mS/cm, but showed a larger relative decrease from its neat-IL value (−73.3% versus −61.7%). This behavior may reflect differences in ion pairing and/or separator compatibility between BF_4_^−^- and TFSI^−^-based systems. Consistent with prior reports [29], the PI gel imbibed with IL-E (run 8) swelled to a greater final thickness than that imbibed with IL-F (run 10).

## 3. Conclusions

A BPDA/ODA/N3300A-based polyimide (PI) gel separator was successfully synthesized and evaluated after imbibing six room-temperature imidazolium-based ionic liquids containing 10 wt% LiTFSI. The resulting IL/LiTFSI-solvated PI gel separators exhibited ionic conductivities exceeding 1 × 10^−3^ S/cm, within the range relevant for the replacement of conventional polyolefin microporous membranes. The PI gel materials exhibited high thermal stability, with onset decomposition temperatures above 561 °C. Among the systems examined, IL-E (EMIM-TFSI) with LiTFSI provided the best overall performance. Ionic conductivity decreased as separator thickness decreased, consistent with the presence of a surface skin and changes in the effective transport pathways. Anion chemistry (TFSI^−^ vs. BF_4_^−^) also influenced ionic conductivity and electrochemical stability in this PI gel separator framework.

The preparation and use of this PI gel were also advantageous in that they allowed us to bypass the time-consuming supercritical CO_2_ extraction needed to create dry aerogels. Another favorable feature of this direct RTIL penetration procedure is the elimination of additional wetting components such as carbonate solvents.

## 4. Materials and Methods

### 4.1. Materials

The diamine 4,4′-oxydianiline (ODA) was purchased from Wakayama Seika Kogyo Co., Ltd., Wakayama, Japan. The dianhydride biphenyl-3,3′,4,4′-tetracarboxylic dianhydride (BPDA) was purchased from UBE America, Inc. The trifunctional cross-linker, triisocyanate Desmodur N3300A, was purchased from Bayer Material Science. N-methylpyrrolidone (NMP) was purchased from Tedia. Acetic anhydride (AA), triethylamine (TEA), lithium bis(trifluoromethanesulfonyl)imide (LiTFSI) salt, Li metal, and polyvinylidene fluoride (PVDF) were all purchased from Sigma-Aldrich. Acetone was purchased from Fisher Chemical. The high-surface-area active carbon powder was purchased from MTI Corporation The Six imidazolium-based RTILs with relatively low viscosities used in the experiments were obtained from IoLiTec, Inc The chemical structures of these ionic liquids and their properties are presented in Table 1. The dianhydride BPDA was vacuum dried at 135 °C overnight prior to synthesis, and the active carbon was dried at 100 °C under vacuum overnight before use. All other reagents were used as received with no further purification.

### 4.2. Preparation of Polyimide Gels

The polyimide gel was formulated as described in our previous investigations [30,37]. The backbone of the polyimide aerogel was synthesized with *n* equivalents of the dianhydride BPDA and *n* + 1 equivalents of the diamine ODA, then cross-linked with the trifunctional isocyanate monomer N3300A to form a three-dimensional network. The polyimide backbone chain length was formulated with *n* = 30 and *n* = 60 between cross-links. The reaction was carried out in the polar aprotic solvent NMP and chemically imidized at room temperature using acetic anhydride (AA) as a water scavenger and triethylamine (TEA) as a catalyst.

The synthesis for *n* = 60, for example, is as follows: BPDA (3.15 g, 10.7 mmol) was added to a solution of ODA (2.18 g, 10.9 mmol) in 38.8 mL of NMP. The mixture was stirred for 15 min until homogeneous. Acetic anhydride (8.10 mL) and TEA (3.0 mL) were added sequentially and stirred for ten minutes. Next, N3300A (0.060 g, 0.119 mmol), dissolved in 5 mL NMP, was added. The mixture was stirred for another 10 min before being cast into a thin film. This thin gel film was then aged for 12–18 h at room temperature in a closed container. The gel film, still in NMP, was then washed with a 50/50 volume–percent mixture of NMP/acetone followed by four subsequent washes with acetone. Both the thin polyimide gel films with *n* = 30 and *n* = 60 were stored in acetone until they were ready for solvent exchange with room temperature ILs or supercritical drying to produce polyimide aerogels.

It is important to reduce the thickness of the separator as much as possible since the thickness of the internal components within a cell has a dramatic effect on the specific energy of the battery. Although the ODA/BPDA/N3300A-based gel film formulation was castable, obtaining thinner films depended strongly on the viscosity of the polyamic acid solution. For example, the viscosity of a cross-linked polyamic acid solution with *n* = 30 remained low for ~10 min after the cross-linker N3300A was added, but increased rapidly as the gelation point was approached, making it difficult to cast a film larger than 2.5 in. × 5 in. Our preliminary work showed that a more controllable viscous solution could be achieved by formulating a gel with a longer chain length, thus limiting the amount of spreading during film casting. With *n* = 60, the viscosity was consistent for at least 15 min after the cross-linking reaction started, providing a larger processing window to produce a one-foot by ten-foot sheet of gel film that was continuously cast on a pilot scale. The resulting PI gel and PI aerogel with *n* of 60 were flexible and foldable, as seen in Figure 3a,b.

### 4.3. Properties of Polyimide Aerogel Films

The polyimide aerogel film was obtained using a supercritical CO_2_ extraction method. Any residual solvent in the aerogel films was removed by further outgassing in an oven at 80 °C overnight under full vacuum. Brunauer–Emmett–Teller (BET) surface area data were obtained from a Micromeritics ASAP 2020 chemisorption apparatus. The morphologies of the aerogel films were examined using a Hitachi S-4700-11 field emission scanning electron microscope. The percent porosity was calculated from the measured bulk density (ρb) and skeletal density (ρs) using Equation (1):

Porosity (%) = (1 − ρb/ρs) × 100%(1)

### 4.4. Preparation of Electrolyte Solvated Polyimide Gels

The ionic liquid (IL) solution with 10 wt% Li salt (0.5289 M) was prepared in a humidity-controlled dry room and mixed at room temperature overnight. The IL/Li salt mixture was then added directly to the acetone-saturated polyimide gel film. The film initially floated on the top layer because of the differences in density and viscosity between the IL/Li salt solution and acetone. The gel film slowly sank to the bottom of the container over a period of one to three days, indicating that the gel was saturated with the electrolyte. The acetone was then removed by allowing the open container to stand in air for 48 h, permitting the acetone to evaporate and leaving only the polyimide gel imbibed with the IL/Li salt electrolyte.

### 4.5. Tensile Test of the PI Gel Film Separator Imbibed with EMIM-TFSI/Li Salt

Tensile properties of the EMIM-TFSI/Li salt solvated PI gel film (batch #6) were compiled in accordance with standard ASTM D882 Tensile Testing of Thin Film & Sheet Plastics. The samples were laser cut. The ends of the specimens were mounted in cardboard tabs and tested on an Instron 4502 test frame, using pneumatic grips to prevent slippage. Samples of two different sizes, with proportional dimensions, were tested at 2 mm/min speed. The data were collected using Bluehill 3 software. Table S1 lists the sample dimensions and tensile property results.

### 4.6. Preparation of Electrodes and Fabrication of Lab Cells

The active carbon, Super P carbon, and PVDF were dry mixed in a weight ratio of 90:5:5. Next, NMP (with PVDF:NMP at ~1:50 by weight) was added to the well-mixed powder. The mixture was stirred at 800 rpm overnight to ensure that the slurry was homogeneous. The prepared slurry was cast on an aluminum foil substrate to a typical thickness of 5 mils. The aluminum foil coated with the slurry was dried at 70 °C under a hood. The remaining NMP was removed in an oven at 110 °C overnight under a full vacuum.

The dried cathode/electrode described above, the Li metal anode, and the polyimide gel imbibed with RTIL/Li salt as a separator were assembled and crimped into lab cells, i.e., coin cells (2325), in an Ar-filled glove box located in a dry room.

### 4.7. Ionic Conductivity Measurement

The ionic conductivity (σ, in S cm^−1^) of the electrolyte-solvated polyimide gels was determined using electrochemical impedance spectroscopy (EIS) at room temperature. The prepared RTIL/Li salt solvated polyimide gel was first cut into 0.5-inch diameter round samples (area A = 1.266 cm^2^). Any electrolyte outside the gel was wiped off using Kimwipes, and then the thickness (L) of the gel film was measured. The gel film was sandwiched between two stainless steel (SS) blocking disks with a diameter of 0.5 inches. Pressure was applied to ensure sufficient contact between the gel film and the two SS electrodes (SS | polyimide gel film | SS). EIS was then measured using a Solartron SI-1287 potentiostat in combination with a Solartron 1255B frequency response analyzer in a frequency range from 10^−1^ to 10^6^ Hz, with a 10 mV AC amplitude perturbation. The data were plotted in Nyquist form, and the overall resistance of the polyimide gel film (R_bulk_) was determined from the intercept of the semicircle with the real axis, Z_real_. The σ of the polyimide gel samples was calculated based on the thickness (L, in cm), surface area (A, in cm^2^), and R_bulk_ (Ω) using Equation (2):

σ = L / (R_bulk_ × A) (2)

## Figures and Tables

**Table 1:** Chemical structure, ionic conductivity, viscosity, and density of ionic liquids. Data obtained from IoLiTec, Inc. technical data sheets.

Type	Chemical Structure	Ionic Conductivity, mS/cm	Viscosity, cP (°C)	Density, g/cm^3^	Decomposition Temperature (°C)
A	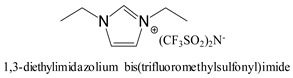	7.27 (20 °C) 8.24 (30 °C)	27.9 (25 °C)	1.45	>200
B	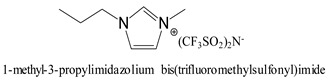	4.40 (20 °C)	56.0 (19 °C)	1.43	N/A
C	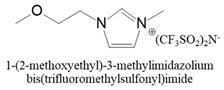	4.81 (30 °C)	46.9 (25 °C)	N/A	N/A
D	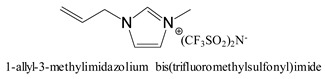	2.76 (25 °C) 8.87 (30 °C)	35.0 (25 °C)	1.49	N/A
E	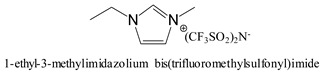	6.63 (20 °C)	39.4 (20 °C)	1.52	250
F	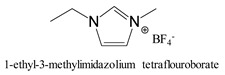	14.1 (25 °C)	33.8 (25 °C)	1.28	N/A

N/A: not available.

## Data Availability

The original contributions presented in this study are included in the article/Supplementary Materials. Further inquiries can be directed to the corresponding authors.

## Supplementary Materials

The following supporting information can be downloaded at: https://www.mdpi.com/article/10.3390/gels12020108/s1. Figure S1. Solid-state ^13^C NMR spectrum of ODA/BPDA/N3300A aerogel with *n* = 30. Figure S2. FTIR spectrum of the ODA/BPDA/N3300A aerogel with *n* = 30. Figure S3. Pore volume distributions as a function of pore diameter for PI aerogels with *n* = 30 and *n* = 60. Table S1. Tensile specimen dimensions and resulting tensile stress at maximum load and Young’s modulus for EMIM-TFSI + 10 wt% LiTFSI-solvated PI gel film separator (*n* = 60). Figure S4. EIS curves of PI gel film, with film thickness of 0.0126 cm and *n* = 60, imbibed with IL-E/Li salt.
