# Supplementary material for: Highly Porous Polyimide Gel for Use as a Battery Separator with Room-Temperature Ionic Liquid Electrolytes
Source: Gels. 2026 Jan 27;12(2):108. doi: 10.3390/gels12020108 (PMC12940053; doi:10.3390/gels12020108)
Supplement: Supplementary file 1 [file gels-12-00108-s001.zip › gels-4055197-supplementary.pdf]

gels

Supplementary Information

Highly Porous Polyimide Gel for Use as a Battery Separator with Room-Temperature Ionic Liquid Electrolytes

Rocco P. Viggiano [1,*], James Wu [1], Daniel A. Scheiman [2], Brianne DeMattia [1], Patricia Loyselle [1] and Baochau N. Nguyen [2,*]

[1] NASA Glenn Research Center, 21000 Brookpark Road, Cleveland, OH 44135, USA; james.j.wu@nasa.gov (J.W.); brianne.t.demattia@nasa.gov (B.D.); patricia.l.loyselle@nasa.gov (P.L.)
[2] Universities Space Research Association, 425 3rd Street SW, Suite 950, Washington, DC 20024, USA; daniel.a.scheiman@nasa.gov
* Correspondence: rocco.p.viggiano@nasa.gov (R.P.V.); baochau.n.nguyen@nasa.gov (B.N.N.)

Shown in Figure S1 is the solid-state ^{13}C NMR spectrum of the ODA/BPDA/N3300A polyimide aerogel with n = 30. The observed resonances are consistent with the functional groups in the cured network. The sharp peak A at ~160 ppm is assigned to imide carbonyls, while the broad peak B corresponds to urea/isocyanurate carbonyls formed during cross-linking. Peak C at ~145 ppm is attributed to aromatic carbons adjacent to the ether linkage in ODA and to the biphenyl bridge in BPDA. Peaks D and E in the range of ~135–115 ppm arise from the remaining aromatic carbons. Broad, low-intensity features (F, ~20–35 ppm) and peak G (~45 ppm) are indicative of methylene carbons from the Desmodur N3300A cross-linker.

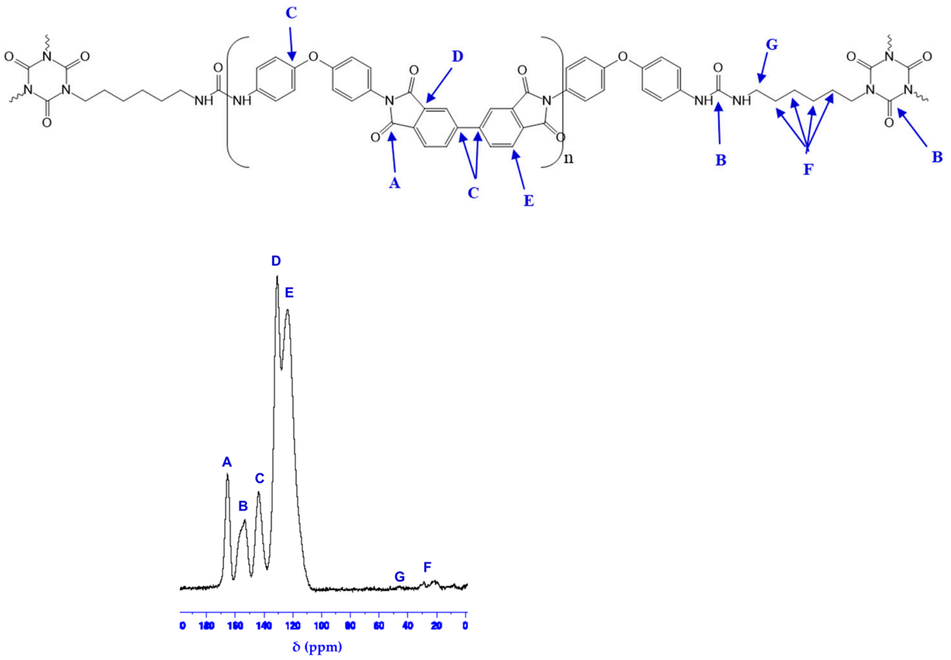

Figure S1. Solid-state ^{13}C NMR spectrum of ODA/BPDA/N3300A aerogel with n = 30.

The chemical structure of the ODA/BPDA/N3300A polyimide aerogel was also characterized by FTIR spectroscopy (Figure S2). The bands at 1780 cm^{-1} and 1718 cm^{-1} (a) are

characteristic of imide C=O stretching vibrations. A strong band at 1244 cm^{-1} (b) corresponds to the C–O–C stretch from ODA. Bands at 1500 cm^{-1} (c) and 1380 cm^{-1} (d) are assigned to aromatic C=C vibrations and C–N stretching, respectively. Bands at 1120 cm^{-1} and 1080 cm^{-1} (e, f), and at 825 cm^{-1} are attributed to aromatic C–H in-plane/out-of-plane bending and aromatic ring vibrations. A very weak, broad band near ~3100 cm^{-1} (g) is consistent with aromatic C–H stretching. The absence of primary amine N–H stretching (3300–3500 cm^{-1}) indicates essentially complete conversion of ODA to the imide/urea-linked network. In addition, the characteristic isocyanate (N=C=O) band (~2270 cm^{-1}) is not observed, consistent with consumption of the triisocyanate (Desmodur N3300A) during cross-linking.

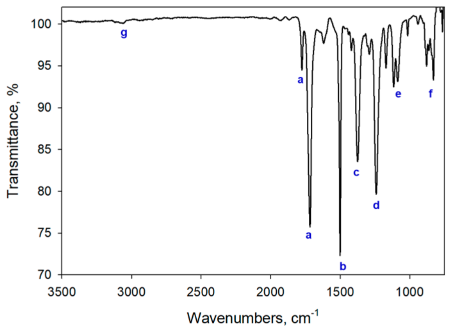

Figure S2. FTIR spectrum of the ODA/BPDA/N3300A aerogel with n = 30.

The BET surface areas of PI aerogels were obtained from gels cast #5 and #6 (Table 2 in the main manuscript). The PI aerogel with n = 30 exhibited a surface area of 419 m^2/g and 91% porosity, higher than the n = 60 aerogel (344 m^2/g and 87% porosity). To further examine this difference, the pore volume distributions as a function of pore diameter are plotted in Figure S3. A bimodal mesopore distribution is observed at 13.3 nm and 17.7 nm for n = 30, and at 11.7 nm and 16.9 nm for n = 60. The n = 30 aerogel exhibits a wider pore-size distribution (approximately 2.2–55 nm), whereas the n = 60 aerogel shows a narrower distribution (approximately 2.3–38 nm). The smaller characteristic pore sizes for n = 60 are consistent with a higher degree of pore collapse during drying, thereby reducing the BET surface area. In addition, the n = 60 aerogel exhibits a larger cumulative pore volume across the measured diameter range.

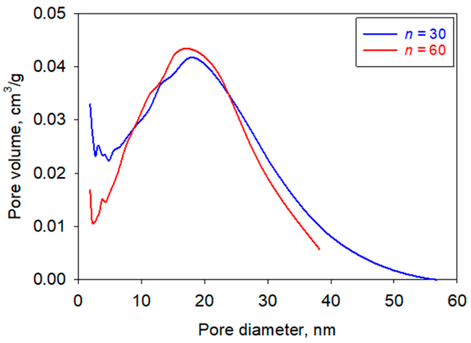

Figure S3. Pore volume distributions as a function of pore diameter for PI aerogels with n = 30 and n = 60.

Table S1. Tensile specimen dimensions and resulting tensile stress at maximum load and Young's modulus for EMIM-TFSI + 10 wt% LiTFSI-solvated PI gel film separator (n = 60).

Sample #	Thickness (mm)	Width (mm)	Length (mm)	Tensile stress at maximum load (Mpa)	Young Modulus (MPa)
1	0.158	12.70	101.60	0.571	26.29
2	0.158	12.70	101.60	0.593	26.21
3	0.158	6.35	50.80	0.596	26.54
4	0.158	6.35	50.80	0.616	26.78
5	0.158	6.35	50.80	0.661	25.87
6	0.158	6.35	50.80	0.627	28.29

Electrochemical impedance spectroscopy (EIS) was used to quantify ionic transport in the ionic-liquid/Li-salt–solvated polyimide (PI) gel separators using a blocking-electrode configuration, consistent with the approach described in the main manuscript. In EIS, a small-amplitude AC voltage perturbation (10 mV) is applied and the complex impedance ($Z = Z' + jZ''$) is measured as a function of frequency. The data are commonly displayed as Nyquist plots ($-Z''$ vs. Z'), where the high-frequency intercept on the real axis corresponds to the bulk electrolyte/gel resistance (Rbulk) for the separator sample. Ionic conductivity (σ) is then calculated from $\sigma = L/(\text{Rbulk}\cdot A)$, where L is the separator thickness and A is the electrode area (Eq 2).

Figure S4 shows representative Nyquist spectra for an n = 60 PI gel film (thickness = 0.0126 cm) imbibed with IL-E/LiTFSI (10 wt% Li salt), measured at room temperature (RT) and after conditioning at 75 °C. The high-frequency intercept shifts to lower Z' at 75 °C (see inset), indicating a decrease in Rbulk and therefore an increase in σ, consistent with the temperature-dependent conductivity trend reported in the manuscript. The low-frequency inclined response reflects electrode polarization/ion-diffusion behavior expected for blocking stainless-steel electrodes and does not affect extraction of Rbulk from the high-frequency region.

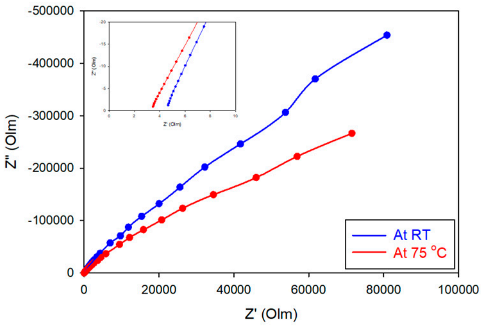

Figure S4. EIS curves of PI gel film, with film thickness of 0.0126 cm and n = 60, imbibed with IL-E/Li salt.
